# Reduction in perforated appendicitis incidence between rural and urban populations after introducing social health insurance in Vietnam: A population‐based study

**DOI:** 10.1002/wjs.12388

**Published:** 2024-11-03

**Authors:** Tran‐Nguyen Nguyen, Yiing‐Jenq Chou, Nicole Huang

**Affiliations:** ^1^ Institute of Public Health College of Medicine National Yang Ming Chiao Tung University Taipei Taiwan; ^2^ Office of People's Committee of Dong Thap Province Cao Lanh Vietnam; ^3^ Office of the Deputy Superintendent National Yang Ming Chiao Tung University Hospital Yilan Taiwan; ^4^ Institute of Hospital and Health Care Administration College of Medicine National Yang Ming Chiao Tung University Taipei Taiwan

**Keywords:** disparity, LMICs, perforated appendix, rural‐urban, Vietnam

## Abstract

**Background:**

Over the years, Vietnam has expanded its social health insurance (SHI) coverage to reduce health disparities. In this population‐based study, we examined the differences in incidences of perforated appendix between rural and urban populations in Vietnam since the expansion of SHI coverage in 2015. We also identified risk factors for perforated appendix in these populations.

**Method:**

The 2015–2019 SHI inpatient claims data from the Social Security Agency of Dong Thap Province were used to elaborate the final sample of 6863 patients aged 18 years or above who underwent an appendectomy. Multivariable probit and logistic regression model were employed to obtain adjusted estimates.

**Results:**

An overall rate of 22.9% for perforated appendix among adults in Dong Thap was revealed. After the expansion of SHI, rural residents consistently had lower rates of perforated appendix than urban residents, but the differences between rural and urban residents were small and decreased over time, that is from 4.4% in 2015 to 3.4% in 2019. Older, male, and poor residents were at a higher risk of perforated appendix in both urban and rural areas. In rural areas, patients with comorbidities, patients who resided in remote communes bordering Cambodia, and patients who had district hospitals or commune facilities as their primary assigned providers were significantly more likely to develop perforated appendix.

**Conclusion:**

Under the SHI in Vietnam, no significant difference was observed in perforated appendicitis incidence between urban and rural residents. Additional efforts are required to reduce poorer outcomes among other high‐risk residents.

## 1 INTRODUCTION

Rural–urban disparities have long been national and international concerns. On a global scale, approximately 56% of rural residents do not have health‐care coverage compared with 22% of urban residents.^1^ This disparity is particularly evident in low‐ and middle‐income countries (LMICs).^2^ Many studies have indicated that expanding health insurance coverage or adopting a universal health‐care system can improve access to health care and improve the health outcomes of the insured population, particularly in rural areas.^3^, ^4^

In 1992, Vietnam, a middle‐income developing country, implemented a social health insurance (SHI) program. To achieve the goal of universal coverage, this SHI program was expanded in 2015. This expansion entailed the implementation of a set of policies regarding the reduction of cost sharing, the elimination of district‐level referral requirements, the expansion of benefit packages, and an increase in provider payments.^5^ Since this expansion, 92% of the entire population of Vietnam has been gradually enrolled into the SHI program (as of 2022).^6^ Most of the currently available studies have evaluated rural and urban disparities in health care by using data collected before 2015 and measures such as out‐of‐pocket expenses, catastrophic health expenditures, and general outpatient or inpatient services.^7^, ^8^ Even studies evaluating the effects of the 2015 reform have focused only on general health services or impoverishment indicators.^9^, ^10^, ^11^ To the best of our knowledge, no study has examined the effects of timely access to surgical care for common diseases such as appendicitis. Hospital admission for perforated appendix is a common indicator of poor access to high‐quality health care, reflecting delayed diagnosis and surgical treatment for appendicitis.^12^ In this population‐based study, we described the trends in perforated appendix in rural and urban areas after the SHI expansion in 2015. We also identified risk factors for perforated appendix in urban and rural areas within the SHI system.

## 2 METHODS

### 2.1 Study settings and data collection

Dong Thap, a province in Vietnam, had a total population of 1,601,300 residents in 2021. About 77% of the total population in Dong Thap Province lived in rural areas.^13^ Of the 30 provincial and district level hospitals in Dong Thap Province, 18 hospitals are located in urban settings and 12 hospitals are located in rural settings. Physician‐ and nurse‐to‐population ratios were 0.93 and 1.49 per 1000 population in 2021.^14^ Surgeons to population ratio was about 5.44 surgeons per 100,000 population in 2019. According to the geographic locations of the hospital providers, patients in either urban or rural settings could access a surgical provider within 30 km (maximum 1‐h riding motorcycle). Data were obtained from SHI inpatient claims for the period between 2015 and 2019 from the Social Security Agency of Dong Thap. Inpatient claims include information on birth year, sex, residential location, enrollment category, dates of hospital admission and discharge, procedure and diagnosis codes, and identifiers of assigned providers and treatment providers.

### 2.2 Sampling and diagnosis of perforated and nonperforated appendix

A total of 7602 patients aged 18 years or more who underwent an appendectomy in Dong Thap between 2015 and 2019 were identified using an adapted version of *International Classification of Diseases, Tenth Revision, Clinical Modification* (ICD‐10‐CM codes: K35, K36, and K37). Only those with either inflamed and unruptured appendix or perforated appendix as their main diagnosis were included in this study. After patients with incomplete information on patient and provider characteristics were excluded, the final sample comprised 6863 patients (Supporting Information S1: Appendix 1). ICD‐10‐CM codes K35.0, K35.1, K35.2, and K35.3 were used to identify perforated appendix. Other cases were referred to as normal appendicitis (Supporting Information S1: Appendix 2).

### 2.3 Classification of rural and urban areas

According to the regulation in Vietnam, areas are classified into three categories from the least urbanized to the most urbanized as follows: communes, townships, and wards (“xã”, “thị trấn”, and “phường” in Vietnamese, respectively).^15^ Patients who resided in communes were referred to as rural residents, and patients who resided in wards and townships were referred to as urban residents. According to the Vietnamese government, a total of eight rural communes in Dong Thap near country borders are designated as remote areas. Therefore, patients living in these eight communes were referred to as remote area residents.

### 2.4 Patient and hospital characteristics

Patient characteristics such as age, sex, socioeconomic status (SES), and comorbidity were examined. In accordance with the SHI scheme, individuals living in poor and near‐poor households and unemployed individuals can receive SHI premium exemptions or subsidies. SES was defined based on an individual's receipt of SHI subsidies. Patients who received SHI premium exemptions or subsidies were referred to as poor, whereas the other patients were referred to as nonpoor (Supporting Information S1: Appendix 3). Charlson comorbidity index (CCI) was used to reflect the patient's health status and constructed as a dichotomous variable: without any comorbidity (CCI = 0) and with at least 1 comorbidity (CCI ≥1). The level of each patient's assigned and treatment providers were also examined. Providers were divided into three levels depending on their capacity and service scope from the least advanced to the most advanced as follows: commune facilities, district hospitals, and provincial hospitals.

### 2.5 Statistical analysis

To adjust for differences in the characteristics of patients, assigned providers, and treatment providers, probit regression models including these variables and interaction terms between the rural area status and year dummies were conducted to determine the differences in the annual trends in the rates of perforated appendix between rural and urban residents from 2015 to 2019. Because our observations can be clustered by providers, we included clustering in our models. Instead of reporting coefficients, we reported the marginal effects of independent variables. We also conducted a subgroup analysis by using logistic regression to identify risk factors for perforated appendix in urban and rural residents. A *p* value of <0.05 was considered statistically significant. All statistical analyses were conducted using SAS 9.4 and STATA 17.0 software packages.

## 3 RESULTS

During the study period, the overall rate of perforated appendix in Dong Thap was 22.9% (Table 1). The proportions of older patients, poor patients, and patients with comorbidities were significantly higher in rural areas than urban areas. A higher proportion of urban patients had provincial hospitals as their primary assigned providers than their rural counterparts. More than 90% of the patients in both rural and urban areas received their appendectomies in province hospitals.

**TABLE 1:** Characteristics of patients with appendicitis in Dong Thap, Vietnam, from 2015 to 2019.

	Overall		Rural		Urban		
	*N*	%	*N*	%	*N*	%	*p* value
Sample size	6863	100	5605	81.7	1258	18.3	
Rupture							
No	5291	77.1	4326	77.2	965	76.7	0.71
Yes	1572	22.9	1279	22.8	293	23.3	
Age							
18‐64	5826	84.9	4720	84.2	1106	87.9	<0.01
≥65	1037	15.1	885	15.8	152	12.1	
Sex							
Male	2850	41.5	2326	41.5	524	41.6	0.92
Female	4013	58.5	3279	58.5	734	58.4	
Socioeconomic status							
Poor	1065	15.5	910	16.2	155	12.3	<0.01
Non‐poor	5798	84.5	4695	83.8	1103	87.7	
Comorbidity							
No (CCI = 0)	5262	76.7	4262	76.0	1000	79.5	0.01
Yes (CCI ≥1)	1601	23.3	1343	24.0	258	20.5	
Level of assigned provider							
Province level	2190	31.9	1454	25.9	736	58.5	<0.01
District level	2850	41.5	2411	43.0	439	34.9	
Commune level	1823	26.6	1740	31.0	83	6.6	
Level of treating provider							
Province level	6346	92.5	5204	92.9	1142	90.8	0.02
District level	517	7.5	401	7.2	116	9.2	
Year							
2015	958	14.0	742	13.2	216	17.2	<0.01
2016	1167	17.0	938	16.7	229	18.2	
2017	1384	20.2	1146	20.5	238	18.9	
2018	1608	23.4	1338	23.9	270	21.5	
2019	1746	25.4	1441	25.7	305	24.2	

### 3.1 Rural–urban differences over time

**FIGURE 1:**
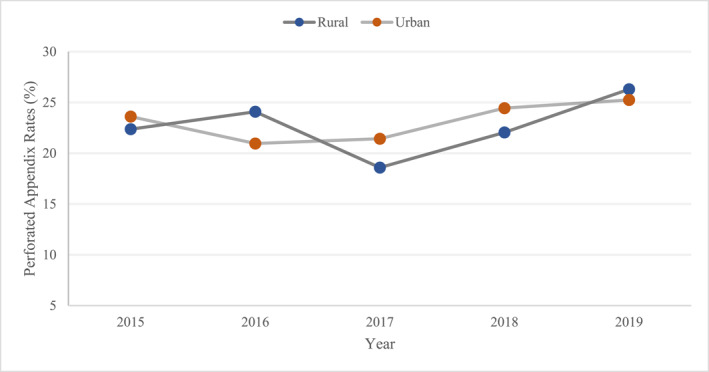
Trends in the rates of perforated appendix by area of residence.

The crude rates of perforated appendix increased in both urban (from 23.6% to 25.3%) and rural areas (from 22.4% to 26.3%) from 2015 to 2019, although few fluctuations were observed (Figure 1). After adjustment, rural residents consistently had lower rates of perforated appendix, and the differences between rural and urban patients decreased over time (Table 2). The magnitudes of the differences were small.

**TABLE 2:** Differences in the adjusted rates of perforated appendix between rural and urban areas over time from 2015 to 2019.

	Marginal effect	*p* value	95% confidence interval	
Year				
2015	−0.044	0.182	−0.109	0.021
2016	−0.042	0.077	−0.088	0.004
2017	−0.039	0.009	−0.069	−0.010
2018	−0.037	0.001	−0.059	−0.015
2019	−0.034	0.050	−0.068	−0.000

*Note*: These results were obtained after patient characteristics (age, sex, SES, and presence of comorbidities), level of assigned provider, level of treatment provider, and clustering for assigned providers were controlled for.

### 3.2 Risk factors for perforated appendix among urban and rural residents

After adjustment, older patients in urban and rural areas had 3.12‐fold (95% confidence interval [CI] = 2.15–4.52) and 2.29‐fold (95% CI = 1.95–2.69) higher risks of perforated appendix compared with their counterparts. Male patients in urban and rural areas had 1.59‐fold (95% CI = 1.21–2.10) and 1.47‐fold (95% CI = 1.30–1.68) higher risks of perforated appendix than female patients (Table 3). Compared with nonpoor patients, poor patients in urban (odds ratio [OR] = 1.72, 95% CI = 1.14–2.60) and rural (OR = 1.21, 95% CI = 1.01–1.46) areas had a significantly higher risk of perforated appendix. In contrast to urban patients, rural patients with comorbidities (OR = 1.17, 95% CI = 1.01–1.35) had a significantly higher risk of perforated appendix compared with those without comorbidities. Compared with patients who resided in nonremote areas, rural patients who resided in remote communes bordering Cambodia (OR = 1.51, 95% CI = 1.15–1.98) had a significantly higher risk of perforated appendix.

**TABLE 3:** Risk factors for perforated appendix among rural and urban residents.

	Rural population		Urban population	
	Crude OR (95% CI)	Adjusted OR (95% CI)	Crude OR (95% CI)	Adjusted OR (95% CI)
Age				
18–64	Ref	Ref	Ref	Ref
≥65	2.41 (2.06–2.81)	2.29 (1.95–2.69)	3.28 (2.30–4.65)	3.12 (2.15–4.52)
Sex				
Female	Ref	Ref	Ref	Ref
Male	1.40 (1.24–1.59)	1.47 (1.30–1.68)	1.44 (1.10–1.87)	1.59 (1.21–2.09)
Socioeconomic status				
Non‐poor	Ref	Ref	Ref	Ref
Poor	1.47 (1.26–1.73)	1.21 (1.01–1.46)	2.21 (1.55–3.15)	1.72 (1.14–2.60)
Comorbidity				
No	Ref	Ref	Ref	Ref
Yes	1.33 (1.16–1.53)	1.17 (1.01–1.35)	1.27 (0.93–1.73)	0.94 (0.67–1.32)
Remote status				
Non‐remote	Ref	Ref		
Remote	1.46 (1.13–1.90)	1.51 (1.15–1.98)	‐	‐
Level of assigned provider				
Province	Ref	Ref	Ref	Ref
District	1.45 (1.23–1.71)	1.62 (1.37–1.92)	1.13 (0.85–1.50)	1.33 (0.98–1.79)
Commune	1.69 (1.42–2.01)	1.42 (1.18–1.72)	1.83 (1.13–2.98)	1.363 (0.78–2.39)
Level of treating provider				
Province	Ref	Ref	Ref	Ref
District	0.16 (0.10–0.26)	0.15 (0.10–0.24)	0.29 (0.15–0.56)	0.273 (0.14–0.54)

Abbreviation: OR, odds ratio.

Although the level of primary assigned provider did not influence the likelihood of experiencing perforated appendix among urban patients, rural patients who had district hospitals (OR = 1.62, 95% CI = 1.37–1.92) or commune facilities (OR = 1.42, 95% CI = 1.18–1.72) as their primary assigned providers were significantly more likely to develop perforated appendix. Compared with patients who received treatment at provincial hospitals, urban (OR = 0.27, 95% CI = 0.14–0.54) and rural (OR = 0.15, 95% CI = 0.10–0.24) patients who underwent their appendectomies at district hospitals were significantly less likely to have perforated appendix.

## 4 DISCUSSION

After the expansion of the SHI system in 2015, the rate of perforated appendix in Dong Thap (22.9%) was relatively lower than the average rates (25.9%) reported in 22 other LMICs.^16^ More specifically, the perforation rate reported in this study was comparable to those reported in some low‐ and middle‐income South Asian countries (Pakistan 20.0%, India 22.0%),^17^, ^18^ but significantly lower than those in African countries (28.5%–57.0%).^19^, ^20^, ^21^, ^22^ It is worth noting that the perforation rate observed in our study was comparable to other neighboring higher‐income countries such as Thailand (21.8%),^23^ China (19.2%),^24^ Taiwan (26.8%),^25^ and South Korea (21.5%),^26^ where these countries also have universal health insurance coverage. More importantly, since this SHI expansion in Vietnam in 2015, no clear disparities have been observed in the risk of perforated appendix between rural and urban residents. During the study period, the rate of enrollment into the SHI program increased from 62% to 86%,^27^ and the out‐of‐pocket expenses of near‐poor individuals decreased to approximately one‐fifth in Dong Thap.^28^ This reform has reduced the financial barriers to prompt diagnosis and treatment for appendicitis in rural areas.^7^ It also presumably increased the availability and quality of health services in rural areas. Some reports revealed a similar level of health service readiness in both rural and urban commune health stations.^14^, ^29^

Compared with rural areas, rates of perforated appendix were slightly higher in urban areas, presumably because of the inappropriate care‐seeking behavior of urban residents. According to a recent review of LMICs, seeking appropriate treatment is delayed because of the following three factors: self‐medication and utilization of alternative drugs, poor awareness of symptoms, and treatment refusal for fear of hospitalization.^16^ Self‐medication, particularly with antibiotics, is a common phenomenon in Vietnam.^30^ In addition, self‐medication and direct purchase of medications from pharmacies without prescriptions are more common in urban areas than in rural areas.^31^ According to a multicenter study, antimicrobial resistance, which results from self‐medication with antibiotics for common infections, is associated with adverse clinical outcomes among patients with acute appendicitis.^32^

Consistent with previous studies in other Asian countries, older age, male sex, and low SES are high‐risk groups of perforated appendix, regardless of the patient's residency.^25^, ^26^ Compared with their counterparts, older and male patients may have a lower perception of pain, which may further delay their hospitalization.^33^ The physiological progress of perforated appendix in older patients may be faster.^33^ In Dong Thap, the absence of family members as caregivers due to seeking employment in neighboring provinces may hinder timely access to care for older residents. Even with health insurance coverage, patients with low SES in urban and rural areas remain disadvantaged in terms of timely access to care, presumably because of disparities in health‐care resources and health literacy.^34^

Consistent with earlier findings in the United States, the diagnosis and treatment of appendicitis may be delayed in rural residents with comorbidities as a result of resource constraints and low quality of care in rural areas.^35^ The accurate and timely diagnosis of appendicitis in the presence of atypical symptoms for common infections of the gastrointestinal tract may be challenging; the lack of advanced equipment and adequate skills among rural providers may further delay treatment among rural residents with other comorbidities.^36^ Chronic conditions such as diabetes may also influence the function of the appendix and adjacent organs, thereby exacerbating the complications of appendicitis.^37^ Another plausible explanation is the low quality of care and the inadequate management of diabetes or chronic conditions in rural areas. Rural residents from communes bordering Cambodia were at a higher risk of perforated appendix than other rural residents. Compared with rural residents from other inland communes, these patients are poorer and are required to travel larger distances to district‐ or province‐level hospitals for treatment.

Furthermore, rural residents who had low‐level providers, such as commune health stations or district hospitals, as their primary source of care were at a higher risk of perforated appendix than their urban counterparts. The availability and quality of medicine, equipment, and treatment and the capabilities of medical staff in diagnosing and treating emergent surgical conditions such as appendicitis may be poorer in rural commune health stations and district hospitals than in provincial hospitals.^38^ By contrast, the capabilities of medical staff in diagnosing and treating appendicitis in urban health stations and district hospitals may be more equivalent to those in provincial hospitals. In Vietnam, because rural areas have poorer service quality and low‐level providers, rural residents tend to seek care outside their residential areas, which presumably leads to a delay in their diagnosis and treatment.^39^

Compared with patients who were treated at district hospitals, those who were treated at provincial hospitals had a significantly higher risk of perforated appendix. This finding has two plausible explanations. First, consistent with the results for high‐income countries, instead of receiving early treatment for appendicitis at the nearest health‐care facility, patients may choose to visit farther higher‐level hospitals, which may delay their treatment and increase the risk of perforated appendix.^36^ Second, because provincial hospitals are better equipped than other service providers, patients with complications or perforated appendix may be referred or transferred to provincial hospitals.

This study has some limitations. First, although we have included CCI to adjust for comorbidities, because of the inherent limitations of administrative data, such as the lack of detailed clinical information, residual confounding may still be possible. Second, the rate of perforated appendix resulting from the misdiagnosis of appendicitis may have been underestimated, thus representing another source of bias. Such underestimation may be either differential or nondifferential between urban and rural areas. No evidence is yet available regarding differential misdiagnosis rates between urban and rural areas in Dong Thap or even in Vietnam. Third, because of the heterogeneity in social, health care, and geographic characteristics, our findings may not be generalizable to other regions in Vietnam.

## 5 CONCLUSION

To the best of our knowledge, this is the first population‐based study to examine the disparities in access to care for appendicitis between rural and urban areas in Vietnam after the 2015 reform of the SHI program. After this reform, the overall rate of perforated appendix did not exceed that reported in neighboring more developed countries and in most of LMICs. In addition, rural residents did not have a higher risk of perforated appendix than their urban counterparts over time. However, older, male, and poor individuals had an increased risk of perforated appendix; thus, these population require further attention. Additional efforts are required to address the inadequate capacity and low quality of care in low‐level health facilities in rural areas and to ensure the availability of medications and medical supplies in low‐level service providers, particularly in commune health stations.

## AUTHOR CONTRIBUTIONS

**Tran‐Nguyen Nguyen**: Conceptualization; data curation; formal analysis; investigation; methodology; resources; validation; visualization; writing—original draft; writing—review & editing. **Yiing‐Jenq Chou**: Conceptualization; investigation; resources; supervision; writing—original draft; writing—review & editing. **Nicole Huang**: Conceptualization; formal analysis; investigation; resources; supervision; validation; writing—original draft; writing—review & editing.

## CONFLICT OF INTEREST STATEMENT

The authors declared no potential conflicts of interest with respect to the research, authorship, and/or publication of this article.

## ETHICS STATEMENT

All authors comply with the journal's ethical policies. This study was granted ethical approval No. NYCU112178AE by Institutional Review Board A of National Yang Ming Chiao Tung University. The secondary data were provided by Social Security Agency of Dong Thap Province and individual identifiers are encrypted for ensuring patient anonymity and confidentiality.

## Supporting information

Supporting Information S1

## References

[1] Scheil‐Adlung, X. 2015. “Global Evidence on Inequities in Rural Health Protection. New Data on Rural Deficits in Health Coverage for 174 Countries.” In ESS Paper Series (ILO), S.P.D. (ILO). Geneva, Switzerland: International Labour Organization.

[2] Strasser, Roger , Sophia M. Kam , and Sophie M. Regalado . 2016. “Rural Health Care Access and Policy in Developing Countries.” Annual Review of Public Health 37(1): 395–412. 10.1146/annurev-publhealth-032315-021507. 26735432

[3] Zogg, Cheryl K. , John W. Scott , Nizar Bhulani , Abbe R. Gluck , Gregory D. Curfman , Kimberly A. Davis , Justin B. Dimick , and Adil H. Haider . 2019. “Impact of Affordable Care Act Insurance Expansion on Pre‐hospital Access to Care: Changes in Adult Perforated Appendix Admission Rates after Medicaid Expansion and the Dependent Coverage Provision.” Journal of the American College of Surgeons 228(1): 29–43.e1. 10.1016/j.jamcollsurg.2018.09.022. 30359835

[4] Huang, N. , W. Yip , H.‐J. Chang , and Y.‐J. Chou . 2006. “Trends in Rural and Urban Differentials in Incidence Rates for Ruptured Appendicitis under the National Health Insurance in Taiwan.” Public Health 120(11): 1055–1063. 10.1016/j.puhe.2006.06.011. 17011602

[5] Le, Quynh Ngoc , Leigh Blizzard , Lei Si , Long Thanh Giang , and Amanda L. Neil . 2020. “The Evolution of Social Health Insurance in Vietnam and its Role towards Achieving Universal Health Coverage.” Health Policy OPEN 1: 100011. 10.1016/j.hpopen.2020.100011. 37383313 PMC10297761

[6] Security, V. S. 2022. “THÔNG TIN BÁO CHÍ: 10 Kết Quả Nổi Bật Của Ngành BHXH Việt Nam Năm 2022. [Press Information: 10 Remarkable Achievements of Vietnam Social Security in 2022].” [cited 2023 01 August]; Available from. https://baohiemxahoi.gov.vn/chidaodieuhanh/pages/thong‐tin‐bao‐chi.aspx?ItemID=19893&CateID=125.

[7] Mao, Wenhui , Yuchen Tang , Tra Tran , Michelle Pender , Phuong Nguyen Khanh , and Shenglan Tang . 2020. “Advancing Universal Health Coverage in China and Vietnam: Lessons for Other Countries.” BMC Public Health 20(1): 1791. 10.1186/s12889-020-09925-6. 33238998 PMC7690086

[8] Matsushima, Midori , Hiroyuki Yamada , and Yasuharu Shimamura . 2020. “Analysis on Demand‐ and Supply‐Side Responses during the Expansion of Health Insurance Coverage in Vietnam: Challenges and Policy Implications toward Universal Health Coverage.” Review of Development Economics 24(1): 144–166. 10.1111/rode.12627.

[9] Nguyen, Chi M. , Mai P. Nguyen , and Lan D. P. Luc . 2023. “How Public Health Insurance Expansion Affects Healthcare Utilizations in Middle and Low‐Income Households: an Observational Study from National Cross‐Section Surveys in Vietnam.” BMC Public Health 23(1): 624. 10.1186/s12889-023-15500-6. 37004009 PMC10067245

[10] Nguyen, Mai P. 2021. “Health Services Utilization Among Older Adults in Vietnam: Evidence from the National Household Living Standard Survey 2016.” Asia‐Pacific Journal of Public Health 34(1): 57–64. 10.1177/10105395211044616. 34486406

[11] Thu Thuong, Nguyen Thi , Yme Van Den Berg , Tran Quang Huy , Do Anh Tai , and Bui Nu Hoang Anh . 2021. “Determinants of Catastrophic Health Expenditure in Vietnam.” The International Journal of Health Planning and Management 36(2): 316–333. 10.1002/hpm.3076. 33022102

[12] AHRQ. Prevention Quality Indicator 02 (PQI 02) Perforated Appendix Admission Rate. 2018 [cited 2023 01 August]; June 2018: Available from. www.qualityindicators.ahrq.gov

[13] General Statistics Office of Vietnam, Statistical yearbook of Vietnam 2021: Statistical Publishing House.

[14] Sở Y tế Đồng Tháp . Báo Cáo Số 511/BC‐SYT Ngày 31 Tháng 12 Năm 2021 Về Tình Hình thực hiện hoạt động y tế năm 2021 và phương hướng, nhiệm vụ năm 2022. 2021: Đồng Tháp.

[15] Ủy ban Thường vụ Quốc hội . 2016. Nghị quyết số 1211/2016/UBTVQH13 ngày 25 tháng 05 năm 2016 về tiêu chuẩn của đơn vị hành chính và phân loại đơn vị hành chính. Hà Nội.

[16] Louw, Johnelize , M. McCaul , R. English , P. S. Nyasulu , J. Davies , C. Fourie , J. Jassat , and K. M. Chu . 2023. “Factors Contributing to Delays to Accessing Appendectomy in Low‐ and Middle‐Income Countries: A Scoping Review.” World Journal of Surgery 47(12): 3060–3069. 10.1007/s00268-023-07183-2. 37747549 PMC10694117

[17] Ahmad, N. , and A. A. Ali . 2020. “Acute Appendicitis Is Still a Morbid Disease in the Developing World.” Pakistan Journal of Medical & Health Sciences 14(3): 537–539.

[18] Paidipelly, Kiran Kumar , and Sangamitra . 2018. “Risk Factors of Acute and Perforated Appendicitis in a Semi‐rural Population: a Retrospective Study.” International Surgery Journal 5(7): 2432. 10.18203/2349-2902.isj20182488.

[19] Zewdu, Dereje , Mekete Wondwosen , Temesgen Tantu , Tamiru Tilahun , Tewodros Teshome , and Ahmed Hamu . 2022. “Predictors and Management Outcomes of Perforated Appendicitis in Sub‐saharan African Countries: A Retrospective Cohort Study.” Ann Med Surg (Lond) 80: 104194. 10.1016/j.amsu.2022.104194. 36045808 PMC9422206

[20] Afenigus, Abebe Dilie , Agumas Mossie Bayieh , and Berhanu Kassahun . 2022. “Treatment Outcomes of Acute Appendicitis and Associated Factors Among Admitted Patients with a Diagnosis of Acute Abdomen in Debre Markos Referral Hospital, Amhara Region, North West Ethiopia.” Journal of Perioperative Practice 32(5): 123–130. 10.1177/1750458920928473. 32638653

[21] Kong, Victor Y. , Bojana Bulajic , Nikki L. Allorto , Jonathan Handley , and Damian L. Clarke . 2012. “Acute Appendicitis in a Developing Country.” World Journal of Surgery 36(9): 2068–2073. 10.1007/s00268-012-1626-9. 22562453

[22] Balogun, OlanrewajuSamuel , Adedapo Osinowo , Michael Afolayan , Thomas Olajide , Abdulrazzak Lawal , and Adedoyin Adesanya . 2019. “Acute Perforated Appendicitis in Adults: Management and Complications in Lagos, Nigeria.” Annals of African Medicine 18(1): 36–41. 10.4103/aam.aam_11_18. 30729931 PMC6380116

[23] Laohawilai, S. , T. Sunthornpinij , and B. Silaruks . 2019. “Risk Factors for Perforated Appendicitis in a Tertiary Hospital.” The Clinical Academia 43(5): 171–178.

[24] Jiang, Li , Zhonghui Liu , Xiaojun Tong , Yang Deng , Jianwen Liu , Xuefei Yang , Fion S. Y. Chan , and Joe K. M. Fan . 2021. “Does the Time from Symptom Onset to Surgery Affect the Outcomes of Patients with Acute Appendicitis? A Prospective Cohort Study of 255 Patients.” Asian Journal of Endoscopic Surgery 14(3): 361–367. 10.1111/ases.12870. 32996273

[25] Lin, K.‐Biao , K. Robert Lai , N.‐Ping Yang , C.‐Lung Chan , Y.‐Hung Liu , R.‐Hao Pan , and C.‐Hsun Huang . 2015. “Epidemiology and Socioeconomic Features of Appendicitis in Taiwan: a 12‐year Population‐Based Study.” World Journal of Emergency Surgery 10(1): 42. 10.1186/s13017-015-0036-3. 26388932 PMC4573493

[26] Lee, Jung Hun , Young Sun Park , and Joong Sub Choi . 2010. “The Epidemiology of Appendicitis and Appendectomy in South Korea: National Registry Data.” Journal of Epidemiology 20(2): 97–105. 10.2188/jea.je20090011. 20023368 PMC3900807

[27] Bảo hiểm xã hội tỉnh Đồng Tháp . Văn bản số: 1486/BHXH‐VP, ngày tháng 9 năm 2020, về việc thực hiện báo cáo tổng kết Nghị quyết số 21‐NQ/TW của Bộ Chính trị Khóa XI. 2020: Đồng Tháp.

[28] Duc Thanh, Nguyen , Bui Thi My Anh , Phung Thanh Hung , Pham Quynh Anh , and Chu Huyen Xiem . 2021. “Impact of Public Health Insurance on Out‐Of‐Pocket Health Expenditures of the Near‐Poor in Vietnam.” Health Services Insights 14: 11786329211017411. 10.1177/11786329211017411. 34093020 PMC8142235

[29] Tran, A. , H. Le Tu , and H. Minh . 2020. “Availability and Readiness of Communal Health Services: Results from 2015 Vietnam District and Commune Health Facility Survey.” International Journal of Healthcare Management 14: 1–7.

[30] Nguyen, Thuy Thi Phuong , Thang Xuan Do , Hoang Anh Nguyen , Cuc Thi Thu Nguyen , Johanna Catharina Meyer , Brian Godman , Phumzile Skosana , and Binh Thanh Nguyen . 2022. “A National Survey of Dispensing Practice and Customer Knowledge on Antibiotic Use in Vietnam and the Implications.” Antibiotics 11(8): 1091. 10.3390/antibiotics11081091. 36009960 PMC9405246

[31] Nga, Do Thi Thuy , Nguyen Thi Kim Chuc , Nguyen Phuong Hoa , Nguyen Quynh Hoa , Nguyen Thi Thuy Nguyen , Hoang Thi Loan , Tran Khanh Toan , et al. 2014. “Antibiotic Sales in Rural and Urban Pharmacies in Northern Vietnam: an Observational Study.” BMC Pharmacol Toxicol 15(1): 6. 10.1186/2050-6511-15-6. 24555709 PMC3946644

[32] Coccolini, Federico , Giuseppe D'Amico , Massimo Sartelli , Fausto Catena , Giulia Montori , Marco Ceresoli , Roberto Manfredi , Salomone Di Saverio , and Luca Ansaloni . 2016. “Antibiotic Resistance Evaluation and Clinical Analysis of Acute Appendicitis; Report of 1431 Consecutive Worldwide Patients: A Cohort Study.” International Journal of Surgery 26: 6–11. 10.1016/j.ijsu.2015.12.063. 26739114

[33] Augustin, Toms , Burt Cagir , and Thomas J. VanderMeer . 2011. “Characteristics of Perforated Appendicitis: Effect of Delay Is Confounded by Age and Gender.” Journal of Gastrointestinal Surgery 15(7): 1223–1231. 10.1007/s11605-011-1486-x. 21557019

[34] Dinh, Ha T. T. , Nguyet T. Nguyen , and Ann Bonner . 2020. “Health Literacy Profiles of Adults with Multiple Chronic Diseases: A Cross‐Sectional Study Using the Health Literacy Questionnaire.” Nursing and Health Sciences 22(4): 1153–1160. 10.1111/nhs.12785. 33034404

[35] Paquette, Ian M. , Randall Zuckerman , and Samuel R. G. Finlayson . 2011. “Perforated Appendicitis Among Rural and Urban Patients: Implications of Access to Care.” Annals of Surgery 253(3): 534–538. 10.1097/sla.0b013e3182096d68. 21209586

[36] Penfold, Robert B. , Deena J. Chisolm , Benedict C. Nwomeh , and Kelly J. Kelleher . 2008. “Geographic Disparities in the Risk of Perforated Appendicitis Among Children in Ohio: 2001‐2003.” International Journal of Health Geographics 7(1): 56. 10.1186/1476-072x-7-56. 18983666 PMC2586023

[37] Panahi, Armon , Venu G. Bangla , and Celia M. Divino . 2023. “Diabetes as a Risk Factor for Perforated Appendicitis: A National Analysis.” The American Surgeon 89(2): 204–209. 10.1177/00031348221124334. 36047489

[38] Thuy, Hua Thanh , Dinh Thu Ha , Phan Van Tuong , Chu Quoc Thinh , and Nguyen Thi Nga . 2021. “Availability of Essential Medicines in Primary Care in Vietnam.” International Journal of Healthcare Management 14(4): 1213–1220. 10.1080/20479700.2020.1756093.

[39] Vuong, Q.‐Hoang , V.‐Phuong La , M.‐Hoang Nguyen , T.‐Huyen T. Nguyen , and M.‐Toan Ho . 2021, Vol. 9. SAGE open medicine. 10.1177/20503121211042512.Good Budget or Good Care: The Dilemma of Social Health Insurance in Vietnam. 20503121211042512. PMC8422827 34504705

