# Supplementary material for: Reduction in perforated appendicitis incidence between rural and urban populations after introducing social health insurance in Vietnam: A population‐based study
Source: World J Surg. 2024 Nov 3;48(12):2964–71. doi: 10.1002/wjs.12388 (PMC11619737; doi:10.1002/wjs.12388)
Supplement: Supplementary file 1 — Supporting Information S1 [file WJS-48-2964-s001.docx]

**Appendix 1:** Sample Selection

^*^From 2015 to 2016, the classification of inpatients and outpatients is nonuniform. The Social Security Agency of Dong Thap uses a length of stay of over 1 day and a treatment expense of over 20 000 VND as criteria to identify inpatient visits. From 2017 to 2019, the classification of inpatients and outpatients is uniform.

^**^According to the Social Security Agency of Dong Thap, the minimum reimbursement for each case is 56% of the total surgical expenditure. The minimum expenditure for appendectomy from 2015 to 2019 is divided into 3 categories:

- From January 2015 to July 2017: In accordance with decision no. 12/2014/QD-UBND on July 22, 2014, by the People’s Committee of Dong Thap, as an amendment to decision no. 28/2012/QD-UBND on August 13, 2012, regarding the prices of healthcare services in the facilities of Dong Thap, the minimum surgical expenditure for appendectomy is 1 060 000 VND, and the minimum reimbursement amount is 56% × 1 060  000 = 593 600 VND.
- From August 2017 to April 2019: In accordance with decision no. 123/2017/NQ-HDND on July 14, 2017, by the People’s Council of Dong Thap regarding the prices of healthcare services not covered for reimbursement by the Health Insurance Fund in Dong Thap, the minimum expenditure for appendectomy is 1 793 000 VND, and the minimum reimbursement amount is 56% × 1 793  000 = 1 004  080 VND. To save medical supplies and equipment during surgical procedures, the Social Security Agency of Dong Thap reimburses appendectomy cases with at least 1 536 857 VND. Therefore, the current minimum reimbursement amount is 56% × 1 536  857 = 860 640 VND.
- From May 2019 to December 2019: In accordance with decision no. 238/2019/NQ-HDND on April 2, 2019, by the People’s Council of Dong Thap regarding the prices of healthcare services not covered for reimbursement by the Health Insurance Fund in Dong Thap, the minimum expenditure for appendectomy is 2 531 000 VND, and the minimum reimbursement amount is 56% × 2 531  000 = 1 417  360 VND. To save medical supplies and equipment during surgical procedures, the Social Security Agency of Dong Thap reimburses appendectomy cases with at least 2 169 429 VND. Therefore, the current minimum reimbursement amount is 56% × 2 169  429 = 1 214  880 VND.

**Appendix 2.** Vietnamese and International Versions of ICD-10-CM

| **Disease code (Vietnamese version)** | **ICD-10-CM (international version)** | **Disease name** | **Classified as perforated appendicitis in this study** |
| --- | --- | --- | --- |
| K35 | K35 | Acute appendicitis |  |
| K35.0 | K35.2 | Acute appendicitis with generalized peritonitis | x |
| K35.1 | K35.33 | Acute appendicitis with peritoneal abscess | x |
| K35.2 | K35.2 | Acute appendicitis with generalized peritonitis | x |
| K35.3 | K35.3 | Acute appendicitis with localized peritonitis | x |
| K35.8 | K35.80 | Acute appendicitis, other and unspecified |  |
| K35.9 | K35.89 | Acute appendicitis, unspecified |  |
| K36 | K36 | Other appendicitis |  |
| K37 | K37 | Unspecified appendicitis |  |

**Appendix 3.** Benefit Groups

| Group code | Benefit Level | Name of Group (English) | Name of Group (Vietnamese) |
| --- | --- | --- | --- |
| CC | 100% | Merit individuals (war veterans before January 1, 1945, and from January 1, 1945, to August 1945), Vietnamese Heroic Mothers, wounded soldiers, sick soldiers | Người có công với cách mạng, hoạt động trước ngày 01/01/1945, từ ngày 01/01/1945 đến 08/1945, Mẹ Việt Nam anh hùng, thương binh, bệnh binh |
| TE | 100% | Children below 6 years of age | Trẻ em dưới 6 tuổi |
| CK | 100% | Other individuals providing meritorious revolutionary services under the provisions of the ordinance on preference treatment | Người có công cách mạng khác (trừ mã CC) |
| CB | 100% | War veterans | Cựu chiến binh |
| KC | 100% | Individuals engaged in resistance and protecting their homeland | Người tham gia kháng chiến |
| BT | 100% | Individuals eligible for monthly social protection benefits within social protection programs for older and disabled individuals | Bảo trợ xã hội hàng tháng |
| HN | 100% | Poor households | Hộ nghèo |
| DT | 100% | Ethnic minorities residing in areas with unfavorable socioeconomic conditions | Dân tộc thiểu số |
| DK | 100% | Individuals residing in areas with extremely unfavorable socioeconomic conditions | Điều kiện kinh tế đặc biệt khó khan |
| XD | 100% | Individuals residing in island communes or districts, in addition to other individuals | Xã đảo, huyện đảo |
| TS | 100% | Relatives of individuals providing meritorious revolutionary services who are parents, spouses, or children of revolutionary martyrs; individuals providing accommodation and supply for martyrs when they were alive. | Thân nhân của người có công với cách mạng là cha đẻ, mẹ đẻ, vợ hoặc chồng, con của liệt sĩ; người có công nuôi dưỡng liệt sĩ. |
| HT | 95% | Individuals eligible for monthly pension or compensation for loss of work capacity | Người hưởng lương hưu, trợ cấp mất sức lao động hằng tháng |
| TC | 95% | Relatives of individuals providing meritorious revolutionary services who are parents, spouses, or children of revolutionary martyrs; individuals providing accommodation for martyrs | Thân nhân có công cách mạng |
| CN | 95% | Near-poor households | Cận nghèo |
| DN | 80% | Employees working in enterprises, nonstate public service providers, and managers or administrators of cooperatives who are eligible for wages or salaries | Người lao động làm trong Doanh nghiệp hoạt động theo Luật doanh nghiệp |
| HX | 80% | Employees working with cooperatives | Người lao động làm trong hợp tác xã theo Luật Hợp tác xã |
| CH | 80% | Employees working in state agencies and sociopolitical organizations | Người lao động làm trong cơ quan Nhà nước, tổ chức Chính trị-xã hội |
| NN | 80% | Employees working in foreign agencies and organizations | Người lao động làm trong cơ quan, tổ chức nước ngoài |
| TK | 80% | Employees working in other organizations | Người lao động làm trong tổ chức khác |
| HC | 80% | Public officials, servants, and employees | Cán bộ, công chức, viên chức |
| XK | 80% | Commune-level part-time cadres | Người hoạt động không chuyên trách ở xã, phường |
| TB | 80% | Individuals who are eligible for monthly social insurance benefits as a result of occupational accidents or occupational diseases and who receive salaries from their employers | Tai nạn lao động, bệnh nghề nghiệp |
| NO | 80% | Employees who take leaves and are eligible for sickness benefits, as indicated by the law of social insurance, as a result of a disease included in the list of diseases requiring long-term treatment issued by the Ministry of Health | Đang hưởng ốm đau, chữa bệnh dài ngày |
| CT | 80% | Individuals who are at least 80 years of age and who are receiving monthly benefits | Người đủ 80 tuổi trở lên đang hưởng trợ cấp tuất hàng tháng |
| XB | 80% | Officials of communes, wards, and townships who quit their jobs and are currently receiving monthly social insurance benefits | Cán bộ xã phường nghỉ việc hưởng trợ cấp bảo hiểm xã hội hàng tháng |
| TN | 80% | Individuals receiving unemployment benefits | Hưởng trợ cấp thất nghiệp |
| CS | 80% | Rubber workers who are receiving monthly benefits in accordance with the regulations of decision No. 206/CP, dated May 30, 1979, regarding workers engaged in heavy and hazardous work and who were forced to quit their jobs because of their age | Công nhân cao su theo Quyết định số 206/CP |
| XN | 80% | Public officers of communes, wards, or townlets who quit their jobs and are currently eligible for monthly social insurance benefits financed through state budget | Cán bộ xã, phường nghỉ việc hưởng trợ cấp từ ngân sách nhà nước |
| MS | 80% | Individuals already terminating entitlement to incapacity benefits, they are currently eligible for monthly benefits financed through state budget | Thôi hưởng mất sức lao động, đang hưởng trợ cấp tháng từ nga |
| HD | 80% | Incumbent national assembly deputies and incumbent members of all people’s councils | Đại biểu quốc hội, Hội đồng nhân dân đương nhiệm |
| TQ | 80% | Relatives of military personnel | Thân nhân quân đội |
| TA | 80% | Relatives of public security workers | Thân nhân công an |
| TY | 80% | Relatives of cryptographic officers | Thân nhân cơ yếu |
| HG | 80% | Individuals donating body organs under the laws of organ donation and transplantation | Hiến tạng |
| LS | 80% | Foreigners studying in Vietnam who received scholarships financed through state budget | Lưu học sinh nước ngoài |
| HS | 80% | Students in primary to secondary school | Học sinh |
| SV | 80% | Students in college and university | Sinh viên |
| GB | 80% | Members of households engaged in the agricultural, forestry, aquacultural, and salt industry who earn an average income in accordance with regulations of the government and the prime minister | Nông, lâm, ngư, diêm nghiệp |
| GD | 80% | Normal households | Hộ gia đình |
| QN | 100% | Military personnel | Quân nhân quân đội |
| CA | 100% | Public security officers | Công an |
| CY | 100% | Cryptographic officers | Công tác cơ yếu |

The following codes are used for the poor socioeconomic group:

- HN, HK: Individuals from poor households
- CN: Individuals from near-poor households
- BT: Individuals receiving monthly social protection benefits
- TN: Unemployed individuals
- DK: Individuals residing in areas with extremely unfavorable socioeconomic conditions

Non-poor socioeconomic group includes the codes of the remaining groups
